# A complex comprising C15ORF41 and Codanin-1: the products of two genes mutated in congenital dyserythropoietic anaemia type I (CDA-I)

**DOI:** 10.1042/BCJ20190944

**Published:** 2020-05-28

**Authors:** Maithili Shroff, Axel Knebel, Rachel Toth, John Rouse

**Affiliations:** MRC Protein Phosphorylation and Ubiquitylation Unit, School of Life Sciences, University of Dundee, Dundee DD1 5EH, U.K.

**Keywords:** complex, congenital dyserythropoietic anaemia type I, disease, nuclease, PD-(D/E)XK

## Abstract

Congenital dyserythropoietic anaemia (CDA) type I is a rare blood disorder characterised by moderate to severe macrocytic anaemia and hepatomegaly, with spongy heterochromatin and inter-nuclear bridges seen in bone marrow erythroblasts. The vast majority of cases of CDA type I are caused by mutations in the *CDAN1* gene. The product of *CDAN1* is Codanin-1, which interacts the histone chaperone ASF1 in the cytoplasm. Codanin-1 is a negative regulator of chromatin replication, sequestering ASF1 in the cytoplasm, restraining histone deposition and thereby limiting DNA replication. The remainder of CDA-I cases are caused by mutations in the *C15ORF41* gene, but very little is known about the product of this gene. Here, we report that C15ORF41 forms a tight, near-stoichiometric complex with Codanin1 in human cells, interacting with the C-terminal region of Codanin-1. We present the characterisation of the C15ORF41–Codanin-1 complex in humans in cells and *in vitro*, and demonstrate that Codanin-1 appears to sequester C15ORF41 in the cytoplasm as previously shown for ASF1. The findings in this study have major implications for understanding the functions of C15ORF41 and Codanin-1, and the aetiology of CDA-I.

## Introduction

Congenital dyserythropoietic anaemia (CDA) represents a heterogeneous group of rare autosomal recessive hereditary disorders characterised by distinct morphological abnormalities of erythroid precursors in the bone marrow, ineffective erythropoiesis and suboptimal reticulocyte count resulting from inadequate marrow response [1,2]. CDA cases have been divided into three major subtypes: CDA-I, CDA-II and CDA-III [3], and CDA variants such as CDA-IV [4]. CDA type II is the most prevalent form of CDA and is characterised by jaundice, hepatosplenomegaly, gallstones, normocytic anaemia and an iron overload [5–7]. This disease is inherited in an autosomal recessive manner, due to mutation in the *SEC23B* gene that encodes for the component of the coat protein complex II (COPII). The coat complex is involved in vesicle trafficking and has two main functions — the physical deformation of the endoplasmic reticulum membrane into vesicles and the selection of cargo molecules for their transport to the Golgi complex [8]. On the other hand, CDA-III is an autosomal dominant disorder that results from mutations in the *KIF23* gene [9]. The protein encoded by this gene is a member of the kinesin-like protein family and plays a critical role during cytokinesis. The clinical manifestations of this type of CDA includes severe erythroid hyperplasia associated with skeletal disorders, mental retardation and hepatosplenomegaly. Similarly, CDA-IV is also an autosomal dominant disorder, caused by mutation in the *KLF1* gene which encodes for a transcription factor involved in the regulation of erythrocyte development [4].

**Figure 1.**
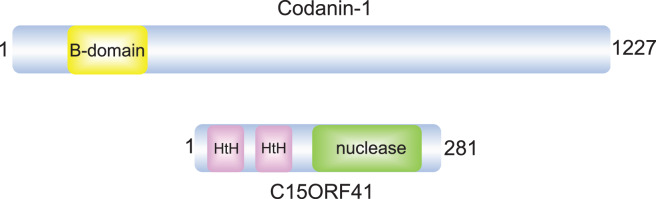
Schematic diagrams of C15ORF41 and Codanin-1. Modular domains are indicated; amino acid numbers are also indicated. HtH, helix-turn-helix.

CDA-I is characterised by moderate to severe macrocytic anaemia, hepatomegaly, and spongy heterochromatin and inter-nuclear bridges in bone marrow erythroblasts. The vast majority (∼80%) of the known cases of CDA type I disease have been found to be associated with mutations in the *CDAN1* gene [4,10,11]. This 28-exon gene encodes for a 134 kDa protein, Codanin-1, which interacts with the histone chaperone ASF1 though a conserved B-domain (Figure 1) [12]. Codanin-1 forms a complex with ASF1, histones H3.1–H4 and Importin-IV in the cytoplasm to regulate histone supply during replication. Codanin-1 is a negative regulator of chromatin replication as it sequesters ASF1 in the cytoplasm, restraining histone deposition and thereby limiting DNA replication [12].

A whole-genome sequencing study identified mutations in the previously uncharacterised locus, *C15ORF41* in CDA-I suggesting that it could be a second causative gene underlying the CDA type I disease [13]. In this study, sequencing and segregation analysis of unrelated CDA-I patients identified two different mutations in *C15ORF41*: a pL178Q substitution altering a highly conserved hydrophobic leucine to a polar glutamine, caused by a T to A transversion in exon 8 of *C15ORF41*; and a pY94C missense mutation that resulted from A to G transition in exon 5 of *C15ORF41*. The *C15ORF41* gene appears to be widely conserved, having orthologs broadly distributed in reptiles, birds and mammals. Prediction of the domain structure of the protein suggest the presence of two N-terminal AraC/XylS-like helix-turn-helix (HtH) domains followed by a PD-(D/E)XK nuclease domain (Figure 1). The PD-(D/E)XK superfamily includes a range of DNA repair nucleases such as MUS81-EME1, FAN1 and XPF-ERCC1 [14]. Furthermore, C15ORF41 shows sequence similarity with archaeal Holliday junction resolvases, such as Hjc [13]; resolvases are DNA repair enzymes which remove Holliday junctions, repair intermediates in which chromatids become topologically intertwined [15]. However, there are no reports of nuclease activity associated with C15ORF41.

To gain clues to the function of C15ORF41, we affinity-purified an epitope-tagged form of the protein from human cells. Here, we report that C15ORF41 forms a tight, near-stoichiometric complex with Codanin-1 in human cells, interacting with the C-terminal region of Codanin-1. We present the characterisation of the C15ORF41–Codanin-1 complex in humans in cells and *in vitro*, and demonstrate that Codanin-1 appears to sequester C15ORF41 in the cytoplasm as previously shown for ASF1.

## Materials and methods

### Cell culture

HEK293 (ATCC) and U2OS FRT Flp-In TRex cells (Invitrogen) were cultured in Dulbecco's Modified Eagle Medium (DMEM) at 37°C in a humidified atmosphere containing 5% CO_2_. All media was supplemented with 10% foetal bovine serum (FBS), penicillin [1% (w/v)], streptomycin [1% (w/v)] and l-glutamine [1% (w/v)]. Additionally, the culture media used to maintain stable cell lines contained hygromycin [100 μg/ml] and blasticidin [7.5 μg/ml].

### Transfection of HEK293 cells

Calcium phosphate transfection was used to generate stable cell lines in HEK293 cells. For each 10 cm dish, two separate reaction mixtures were prepared — (a) 0.5 ml of 2× HEPES buffered saline (HBS) (280 nM NaCl, 1.5 mM NaHPO_4_·7H_2_O, 50 mM HEPES pH 7.0) and (b) transfection mixture containing DNA (10 μg — containing 9 μg of the Flp recombinase vector, pOG44 and 1 μg of the cDNA vector), CaCl_2_ (2 M, 61 μl) made up to a total volume of 0.5 ml using sterile H_2_O. The transfection mixture was then added drop-wise to the HBS tubes with gentle shaking to ensure sufficient aeration. This was done to make sure that the calcium phosphate-DNA precipitate is as fine as possible as this enhances transfection efficiency. The resulting mixture was then added on top of the cells in a drop-wise manner.

### Stable transfection of U-2-OS FlpIn TREX cells

Transfection was carried out using GeneJuice transfection reagent as per the manufacturer's instructions (Merck Millipore). For each 10 cm dish of U2OS FlpIn TREX cells (Invitrogen), transfection mix containing serum-free medium (OPTIMEM, GIBCO) (1 ml) and GeneJuice (60 μl) was prepared. DNA (10 μg — containing 9 μg of pOG44 and 1 μg of the cDNA vector) was then added to this and mixed gently. The resulting mixture was then added drop-wise on the cells. Successful integration was selected using medium supplemented with hygromycin and blasticidin. The expression of the transfected clones was verified by inducing the cells at different concentrations of tetracycline 24 h prior to lysis.

### Antibodies

**Figure 2.**
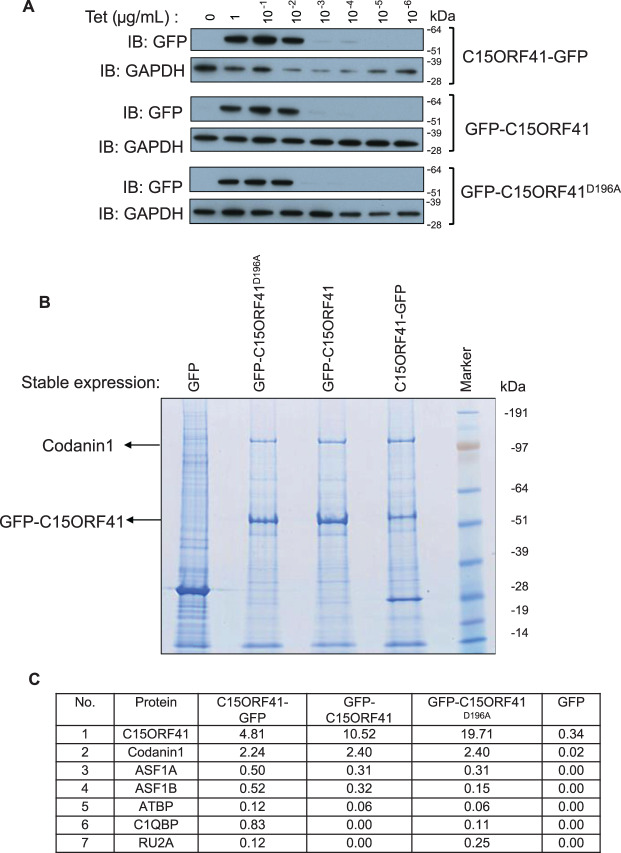
Identification of proteins interacting with C15ORF41. (**A**) Human C15ORF41 with a GFP tag at either the N-terminus (‘GFP-C15ORF41’) or C-terminus (‘C15ORF41-GFP’), or C15ORF41^D196A^ with a GFP tag at the N-terminus, was inserted by recombination at the FRT sites of HEK293 Flp-In™ TREx™ cells. After incubation of cells for 16 h with the tetracycline (Tet) at the indicated concentrations, extracts (20 μg) were subjected to SDS–PAGE and western blotting with the antibodies indicated. (**B**) Immunoprecipitation of GFP-tagged forms of C15ORF41, using GFP-Trap affinity beads, was carried out from ∼40 mg of cell lysate from HEK293 cells stably expressing the indicated proteins. Cells expressing GFP only were used as a control. Anti-GFP precipitates were subjected to SDS–PAGE followed by staining with colloidal Coomassie Blue. (**C**) Table listing C15ORF41-interacting proteins with corresponding emPAI scores [16] for the individual proteins interacting with C15ORF41.

**Figure 3.**
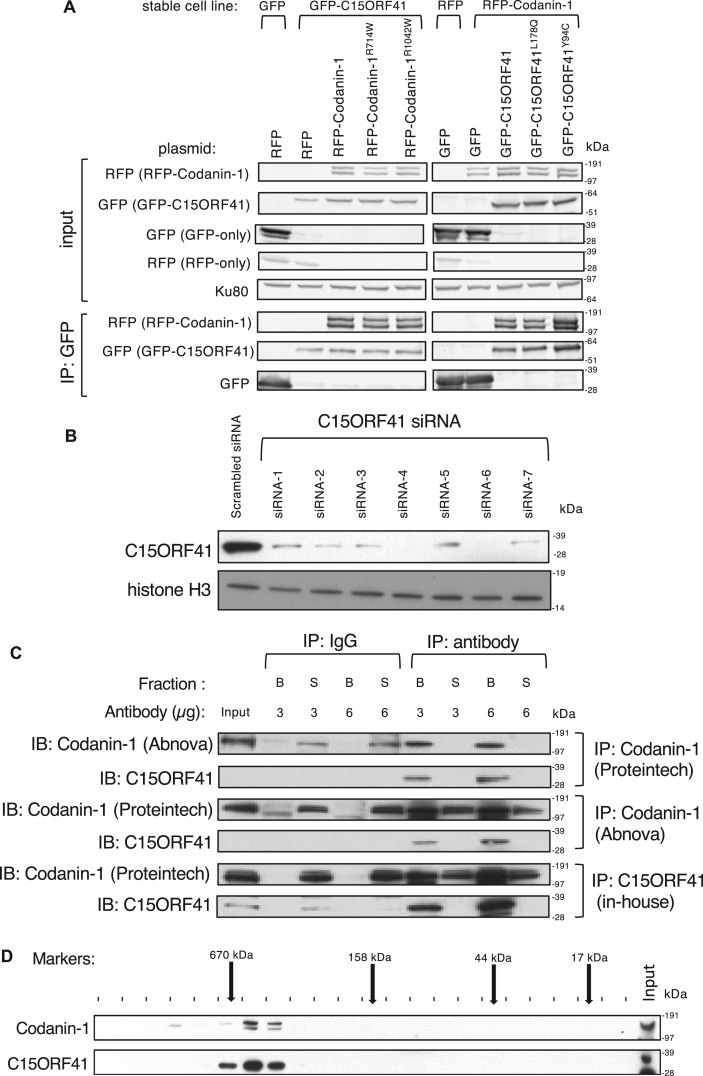
Validation of the C15ORF41–Codanin-1 interaction. (**A**) U2OS cells stably expressing GFP or GFP-tagged C15ORF41 were transfected with plasmids expressing RFP only, RFP-tagged Codanin-1 or RFP-tagged Codanin-1 bearing the pathogenic mutations indicated. In parallel, U2OS cells stably expressing RFP or RFP-tagged Codanin-1 were transfected with plasmids expressing GFP only, GFP-tagged C15ORF41 or GFP-tagged C15ORF41 bearing the pathogenic mutations indicated. Lysates (∼0.5 mg) were subjected to immunoprecipitation with GFP-Trap affinity beads, and precipitates were subjected to SDS–PAGE and western blotting using antibodies indicated (lower panels). The upper panels show western blotting of the input cell extracts. (**B**) U2OS cells were transfected with several different C15ORF41-specific siRNAs (100 nM) as indicated. After 24 h, extracts were prepared and subjected to western blotting with the antibodies indicated. (**C**) Extracts of U2OS cells were subjected to immunoprecipitation with the indicated amounts of two different commercial antibodies against Codanin-1, or in-house antibodies against C15ORF14 conjugated to protein G-sepharose. Beads (B) and supernatants (S) were boiled in LDS sample buffer and subjected to SDS–PAGE and western blotting with the antibodies indicated on the left of the panels. A non-specific (anti-GFP) IgG was used as control. (**D**) Whole-cell HEK293 cell extracts were subjected to size exclusion chromatography using a Superdex 200 16/60 column. Alternate fractions were subjected to SDS–PAGE gel followed by western blot using antibodies against C15ORF41 (in-house) and Codanin-1 (Abcam). The elution position of molecular mass markers is indicated by the arrows above.

The antibodies used in the study included GAPDH (14C10, Cell signalling), Ku80 (ab3107, Abcam), RFP (ab62341, Abcam), Codanin-1 (17597-1-AP, Proteintech and H00146059-M01, Abnova). The antibodies for GFP (S268B) and C15ORF41 (S722D) were generated by the Division of Signal Transduction Therapy at the University of Dundee in sheep. Secondary HRP conjugated antibodies (anti-sheep #31480, anti-rabbit #31460 and anti-mouse #31430) were purchased from Pierce. Secondary fluorescent antibodies were used in Figure 2A and Figure 3: donkey anti-sheep Alexa-Fluor 680 #A21102 from Thermo Fischer and donkey Alexa-Fluor anti-rabbit 800 #926-32213 from LiCOR.

### Production of C15ORF41 antibodies

GST-C15ORF41 expressed in bacteria using plasmid DU49143 was emulsified in Freund's adjuvant prior to injection. Antisera were raised in sheep at the Scottish National Blood Transfusion Service (Penicuik, U.K.). As a standard protocol, one pre-immune bleed was taken on the day of the first injection of the antigen. At least three more injections, one every 28 days, were administered and blood was taken seven days after the second, third and fourth injections. To yield a fourth bleed of the antibody this was repeated once more. Typically 750 ml of blood was taken per bleed, which yields 250–350 ml of serum. Each bleed was allowed to clot overnight at 4°C and, following centrifugation for 60 min at 1500×***g*** at 4°C, the sheep antiserum was decanted though glass wool and stored at −20°C. For purification, the serum was heated for 20 min at 56°C followed by filtration though a 0.45 µM filter. The antiserum was diluted 1 : 1 in 50 mM Tris/HCl pH 7.5 with 2% Triton X-100 and anti-GST antibodies were depleted using a column of GST coupled to activated CH Sepharose. Flow-through fractions were affinity-purified against the relevant antigen. The antibody was eluted with 50 mM glycine pH 2.5 and neutralised by dialysing overnight into PBS. The sheep number is S722D, and it is the 3rd bleed that was used in this study.

### Plasmids

All DNA constructs used in this study, with one exception,^1^ were generated in-house and verified by DNA sequencing on both strands. DNA for bacterial protein expression was transformed into *E. coli* 21 (Merck). All cDNA plasmids generated for this study are listed on the table below. All plasmids are available, with datasheets describing the insert sequence and how they were made, on request from our Reagents division (https://mrcppureagents.dundee.ac.uk/reagents-cdna-clones).

**Table:** 

Plasmid	Vector	Ref.
pcDNA5 FRT/TO GFP	Mammalian	DU13156
pcDNA5D FRT/TO C15ORF41 GFP	Mammalian	DU49145
pcDNA5D FRT/TO GFP C15ORF41	Mammalian	DU49166
pcDNA5D FRT/TO GFP C15ORF41^D196A^	Mammalian	DU49180
pcDNA5D FRT/TO GFP 3C C15ORF41	Mammalian	DU49952
pcDNA5D FRT/TO GFP 3C C15ORF41^D196A^	Mammalian	DU49953
pcDNA5D FRT/TO GFP 3C C15ORF41^Y94C^	Mammalian	DU50092
pcDNA5D FRT/TO GFP 3C C15ORF41^L178Q^	Mammalian	DU50093
pcDNA5D FRT/TO RFP 2A GFP 3C C15ORF41	Mammalian	DU50173
pcDNA5D FRT/TO NLS CDAN1 TEV RFP 2A 2A GFP 3C NLS C15ORF41	Mammalian	DU50443
pcDNA5D FRT/TO NLS CDAN1^Q1000-S1227^ TEV RFP 2A 2A GFP 3C NLS C15ORF41	Mammalian	DU50585
pcDNA5D FRT/TO NLS CDAN1^M1-T200^ TEV RFP 2A 2A GFP 3C NLS C15ORF41	Mammalian	DU50593
pcDNA5D FRT/TO NLS CDAN1^P800-S1227^ TEV RFP 2A 2A GFP 3C NLS C15ORF41	Mammalian	DU50594
pcDNA5D FRT/TO NLS CDAN1^T200-S1227^ TEV RFP 2A 2A GFP 3C NLS C15ORF41	Mammalian	DU50622
pcDNA5D FRT/TO NLS CDAN1^M1-C400^ TEV RFP 2A 2A GFP 3C NLS C15ORF41	Mammalian	DU50623
pcDNA5D FRT/TO NLS CDAN1^M1-P800^ TEV RFP 2A 2A GFP 3C NLS C15ORF41	Mammalian	DU50624
pcDNA5D FRT/TO NLS CDAN1^M1-Q1000^ TEV RFP 2A 2A GFP 3C NLS C15ORF41	Mammalian	DU50625
pcDNA5D FRT/TO NLS CDAN1^C400-S1227^ TEV RFP 2A 2A GFP 3C NLS C15ORF41	Mammalian	DU50630
pcDNA5D FRT/TO NLS CDAN1^M1-G600^ TEV RFP 2A 2A GFP 3C NLS C15ORF41	Mammalian	DU50632
pcDNA5D FRT/TO NLS CDAN1^G600-S1227^ TEV RFP 2A 2A GFP 3C NLS C15ORF41	Mammalian	DU50633
pNIC28 Bsa4 6His Tev C15ORF41^V18M C223S W237R^	Bacterial	DU63514
pNIC28 Bsa4 6His Tev C15ORF41^V18M D196A C223S W237R^	Bacterial	DU61049
pcDNA5D FRT/TO RFP 3C CDAN1	Mammalian	DU50088
pcDNA5D FRT/TO RFP 3C CDAN1^R714W^	Mammalian	DU50105
pcDNA5D FRT/TO RFP 3C CDAN1^R1042W^	Mammalian	DU50107
pET28a 6His z-basic TEV CDAN1^S1000-S1227^	Bacterial	DU63118
pET28a 6His z-basic TEV CDAN1^P800-S1227^	Bacterial	DU63119
pET28a 6His z-basic TEV CDAN1^S840-S1227^	Bacterial	DU63120

### Small interfering RNA

A list of the seven C15ORF41-specific siRNAs are listed below, all from

**Table:** 

C15ORF41 siRNA ♯1	GCAUGCCAUUGGUCAUGAG(dTdT)
C15ORF41 siRNA ♯2	ACCUGUCCUUCCUAGAUGA
C15ORF41 siRNA ♯3	CCUGUCCUUCCUAGAUGAA
C15ORF41 siRNA ♯4	ACAUCAUACUUCGGAAGCA
C15ORF41 siRNA ♯5	AACCUGUCCUUCCUAGAUG
C15ORF41 siRNA ♯6	GUACCAGUUGCUGUAGAAG
C15ORF41 siRNA ♯7	AAUCAGGUCUAUCAGUGCA

### Cell lysis

Cells were lysed in ice cold standard lysis buffer: 50 mM Tris–HCl pH 7.5 plus 150 mM NaCl, 270 mM sucrose, 1% (v/v) Triton X-100 and 0.5% (v/v) NP40 with protease inhibitor cocktail (Roche) and 0.5 U/ml benzonase, or RIPA buffer: 50 mM Tris–HCl, pH8.0, 150 mM NaCl, 5 mM EDTA, 1% (v/v) NP40, 0.5 mM sodium deoxycholate, 0.1% SDS with protease inhibitor cocktail (Roche) and 0.5 U/ml benzonase. Lysates were incubated on ice for 30 min prior to clarification.

### Immunoprecipitation of endogenous proteins

0.5 ml of cell lysate (0.5 mg of protein) was incubated for 2–3 h on a rotating platform at 4°C with 1–3 μg of antibody. Washed Protein G-Agarose (10 μl of beads per 1 μg of antibody) were added and incubated for a further 45 min. The beads were collected by magnetic separation, the supernatant was discarded, and the beads were washed twice with 1 ml of lysis buffer and once with 1 ml of 10 mM Tris/HCl (pH 8.0). Finally, beads were denatured in 2× LDS sample buffer (9 : 10 : 1 4× LDS sample buffer: 500 µl sterile water: 50 µl β-ME) and boiled at 95°C for 5 min. The samples were filtered for any beads using the Costar Spin-X centrifuge tube filters and the resulting immunoprecipitates were analysed by SDS–PAGE and immunoblotting.

### Mass spectrometry

Immunoprecipitates were denatured in LDS sample buffer (Invitrogen) supplemented with 0.1 M DTT, incubated for 1 h at 37°C and then heated for 5 min at 95°C. Samples were filtered through a Spin-X column by centrifugation, resolved by 4–12% gradient SDS–PAGE, and stained with colloidal Coomassie blue overnight. Gel lanes were cut into slice, and each slice was washed sequentially with water, 50% acetonitrile (v/v), 0.1 M NH_4_HCO_3_ and 50% acetonitrile (v/v)/50 mM NH_4_HCO_3_ until the gel pieces were colourless. All washes were performed for 10 min on a shaking platform. Proteins were then reduced with 10 mM DTT/0.1 M NH_4_HCO_3_ at 65°C for 45 min and alkylated with 50 mM Iodoacetamide/0.1 M NH_4_HCO_3_ for 20 min at room temperature. They were then washed with 50 mM NH_4_HCO_3_ and 50 mM NH_4_HCO_3_/50% acetonitrile (as before). Gel pieces were shrunk with acetonitrile for 15 min. Gel pieces were then rehydrated and proteins were digested in 50 mM triethylammonium bicarbonate containing 5 µg/ml trypsin at 30°C for 16 h on a shaker. An equivalent volume of acetonitrile (same as trypsin) was added to each sample and further incubated on a shaking platform for 15 min. The extracted supernatant was stored in a low-binding Eppendorf tube and dried by Speed-Vac. The samples were stored at −20°C prior to peptide extraction and injection into the mass-spectrometer. The mass spectrometric analysis was conducted by data-dependent acquisition with spectra acquired by collision-induced dissociation on an LTQ Orbitrap Velos. Data were analysed using Mascot and Scaffold. The mass spectrometry results are reported as an exponentially modified protein abundance index (emPAI) as it takes into account the number of theoretical peptides of different proteins and normalises the values accordingly [16].

### SDS–PAGE and immunoblotting

Samples were mixed with NuPAGE LDS sample buffer (Thermofisher) containing 5% (v/v) 2-mercaptoethanol, boiled at 95°C for 5 min, resolved by SDS–PAGE (4–12% NuPage gradient gel, Thermofisher) using MOPS buffer and transferred on to 0.45 μm nitrocellulose membranes (GE Life Sciences). Membranes were blocked with PBS-T buffer (PBS, 0.1% Tween-20) containing 5% (w/v) non-fat dried skimmed milk powder (PBS-TM) at room temperature for 1 h. Membranes were subsequently probed with the indicated antibodies in PBS-T containing 5% (w/v) bovine serum albumin (BSA) at the indicated concentrations. After probing with the relevant secondary antibody, the membranes were then analysed using either chemiluminescence or using NIR fluorescent detection on a Li-COR Odyssey CLx imager. In Figure 3, filters were incubated simultaneously with in-house sheep anti-GFP antibodies and rabbit anti-RFP antibodies. After washing filters were incubated simultaneously with the secondary antibodies donkey anti-sheep Alexa-Fluor 680 (red) and donkey Alexa-Fluor anti-rabbit 800 (green).

### Size exclusion chromatography

Around 20 mg of total cell extract from HEK293 cells was loaded onto the Superdex 200 16/60 column, which was equilibrated in gel filtration buffer (50 mM Tri pH 7.5, 150 mM NaCl, 5% glycerol, 1 mM DTT, 0.03% (v/v) Brij 35). 1.5 ml fractions were collected and subsequently analysed by western blotting.

### Recombinant protein expression and purification from *E. coli*

Expression of wild-type and mutant His_6_-tagged C15ORF41 and His_6_-tagged Codanin-1-C was induced using IPTG (100 mM and 250 mM, respectively) at 16 °C overnight. The cells were collected and lysed in 50 mM Tris pH 7.5, 250 mM NaCl, 30 mM Imidazole, 1 mM DTT, 1 mM Pefebloc, 10 μg/ml leupeptin, supplemented with 250 mM NaCl and 5% (v/v) glycerol after sonication. The lysates were clarified and the proteins purified over Ni^2+^-NTA agarose using standard protocols. The proteins were then subjected to dialysis overnight (buffer: 50 mM Tris–HCl pH 7.5, 500 mM NaCl, 5% (v/v) glycerol, 1 mM DTT). Codanin-1-C was subjected to in solution cleavage (during dialysis) using His-TEV protease. The cleaved His-tag and the protease were sequestered using Ni^2+^-NTA agarose. Purified C15ORF41 was once again immobilised on Ni^2+^-NTA agarose and the beads were subsequently incubated with an excess of purified Codanin-1-C. The complex was eluted using 400 mM imidazole and subjected to size exclusion chromatography using a Superdex 200 16/60 column into storage buffer (50 mM Tri pH 7.5, 500 mM NaCl, 5% (v/v) glycerol, 0.5 mM TCEP) and kept at −80 °C.

## Results

### Mass-spectrometric identification of C15ORF41-interacting proteins

To obtain clues to the function of C15ORF41, we screened for C15ORF41-binding partners. To this end, we generated HEK293 stable cell lines expressing GFP-tagged forms of C15ORF41, using the Flp-In™ T-REx™ system which allows stable, tetracycline (Tet)-inducible expression of a gene of interest from a specific genomic location. Cells expressing C15ORF41 (wild-type) tagged with GFP at either the C-terminus (C15ORF41-GFP) or the N-terminus (GFP-C15ORF41), or a mutant form of N-terminally GFP-tagged C15ORF41 (D^196^A) in which the conserved Asp residue at position 196 was mutated to alanine, were generated. Residue 196 lies within the active site of the enzyme and substituting the aspartic acid at this position to alanine should render the enzyme catalytically inert. A control cell line in which the GFP-tag alone was stably expressed under the influence of a tetracycline promoter was also used. Dose-dependent tetracycline-inducible expression of the GFP-tagged C15ORF41 proteins was observed (Figure 2A).

To identify proteins interacting with C15ORF41, GFP-Trap resin was used to immuno-purify the GFP-tagged C15ORF41 fusion proteins shown above. As shown in Figure 2B, when the GFP precipitates were subjected to SDS–PAGE, a band of the expected molecular mass for GFP-tagged C15ORF41 (∼55 kDa) was observed from extracts of the relevant cell lines, but not when cells expressing GFP only were used. Strikingly, we observed an intensely-staining band co-purifying with GFP-C15ORF41 regardless of whether the GFP tag was at the N- or C-terminus, and also with the C15ORF41 (D^196^A) mutant. This band corresponds to an endogenous protein, migrating between the 97 and 191 kDa markers. No such band was observed with GFP-only. To identify this protein, as well as other potential C15ORF41 interactors, the protein gels were cut into slices, and proteins were extracted and subjected to tryptic digestion prior to analysis by liquid chromatography tandem mass spectrometry (LC-MS/MS) (Figure 2C). Strikingly, the endogenous protein co-purifying with GFP-C15ORF41 was identified as Codanin-1, the product of the gene most commonly mutated in CDA-I. The other C15ORF41-interacting proteins identified included ASF1A and ASF1B, but it is likely that the interaction between C15ORF41 and ASF1 is bridged by Codanin-1 [12].

### Validating the interaction of C15ORF41 with Codanin-1

To validate the interaction between C15ORF41 and Codanin-1, we first tested the association of epitope-tagged forms of these proteins co-expressed in human cells. U2OS cells stably expressing GFP-C15ORF41 (or GFP only) were transfected with plasmids encoding red fluorescent protein (RFP)-tagged Codanin1 or RFP-only. As shown in Figure 3A, RFP-Codanin-1 was detected in anti-GFP precipitates from cells co-expressing GFP-C15ORF41 and RFP-Codanin-1, but not in GFP precipitates from extracts of cells co-expressing GFP-only and RFP-Codanin-1 (Figure 3A). We also tested the impact of two pathogenic *C15ORF41* mutations and two pathogenic *CDAN1* mutations on the C15ORF41–Codanin1 interaction. *CDAN1* mutations R714W or R1042W [11,17–19] did not affect the interaction of Codanin-1 with C15ORF41, at least under these conditions. Moreover, these pathogenic *C15ORF41* mutations L178Q and Y94C [13] had no apparent effect on the interaction with Codanin1, at least under the conditions used here (Figure 3A). Thus, the pathogenic impact of the mutations tested cannot be explained by disruption of the C15ORF41–Codanin1 interaction, but we cannot exclude the possibility than other pathogenic *C15ORF41* or *CDAN1* mutations disrupt the interaction. We tested the salt resistance of the interaction between tagged forms of C15ORF41 and Codanin-1, and found that the interaction between GFP-C15ORF41 and RFP-Codanin-1 was able to withstand salt concentrations up to 1 M even in RIPA buffer containing 0.1% SDS, suggesting a robust interaction (Supplementary Figure S1).

We next wished to test the interaction between the endogenous proteins, and to this end, we raised antibodies against full-length human C15ORF41 (fused to GST) expressed in bacteria. As shown in Figure 3B, these antibodies recognise a band of the expected molecular mass for C15ORF41 in extracts of HEK293 cells, and band intensity was reduced when cells were transfected with a range of different C15ORF41-specific siRNAs, demonstrating that the major band recognised by these antibodies corresponds to C15ORF41. In immunoprecipitation experiments, Codanin-1 was detected in anti-C15ORF41 immunoprecipitates, and C15ORF41 was detected in anti-Codanin-1 immunoprecipitates, using two commercially available Codanin-1 antibodies (Figure 3C). We also analysed the C15ORF41–Codanin1 complex by size exclusion chromatography, and found that the two proteins co-elute when extracts of HEK293 cells was applied to a Superdex 200 column (Figure 3D). The C15ORF41–Codanin-1 complex elutes close to the 670 kDa molecular mass standard. A 1 : 1 interaction stoichiometry would result a complex of ∼166 kDa, suggesting that the complex might oligomerize, although shape effects cannot be excluded. It is also possible that the elution position is influenced by other proteins in the complex, such as ASF1A and ASF1B. Taken together, these experiments confirm the interaction between C15ORF41 and Codanin-1, and that pathogenic mutations in either subunit appear not to affect the formation of the C15ORF41–Codanin-1 complex.

### Defining the C15ORF41-interacting domain on Codanin-1

**Figure 4.**
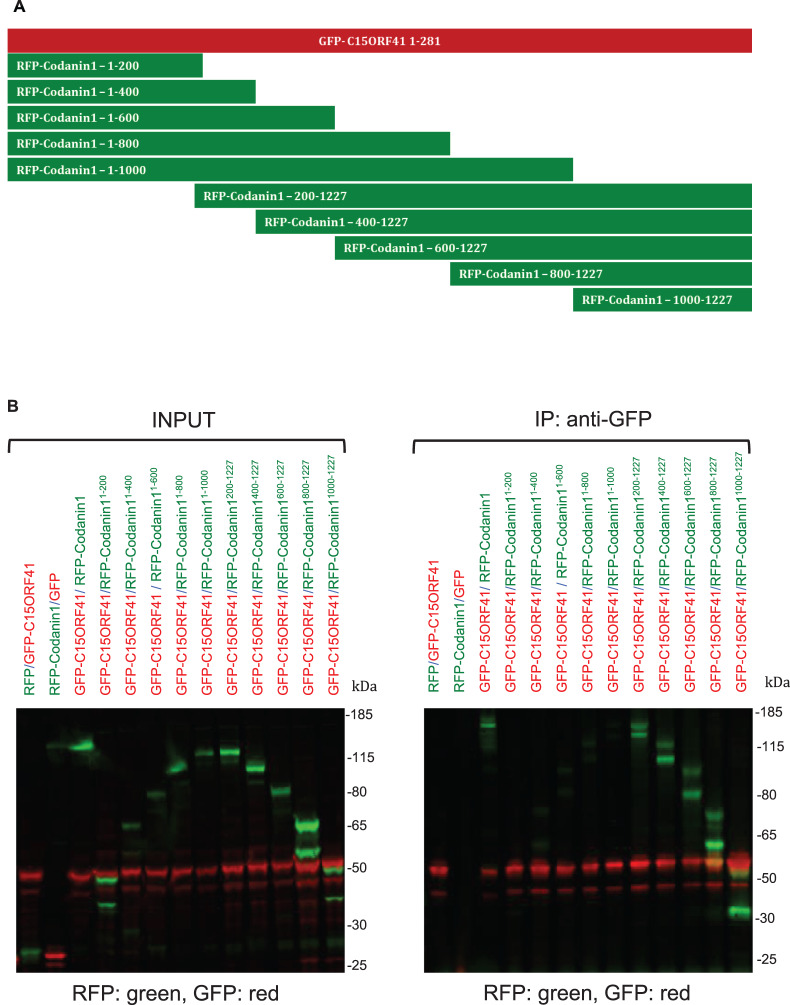
Defining the domain on Codanin-1 interacting with C15ORF41. (**A**) Schematic representation of the Codanin1 deletion constructs for identifying the interaction domain between C15ORF41 and Codanin-1. (**B**) U2OS cells were transiently transfected with bicistronic vectors co-expressing the full-length GFP-C15ORF41 (or GFP only), and full length or truncated versions of RFP-tagged Codanin-1 (or RFP only). Cell extracts were subjected to immunoprecipitation with GFP-Trap beads; precipitates were subjected to SDS–PAGE followed by western blotting (right panel). The membrane was incubated simultaneously with in-house sheep anti-GFP antibodies and rabbit anti-RFP antibodies. After washing filters were incubated simultaneously with the secondary antibodies donkey anti-sheep Alexa-Fluor 680 (red) and donkey Alexa-Fluor anti-rabbit 800 (green). Consequently, GFP-tagged proteins (GFP-tag or GFP-C15ORF41) are represented by red bands, whereas RFP-tagged proteins (RFP-tag or RFP-Codanin-1) are represented by green bands. GFP-C15ORF41/RFP and GFP/RFP-Codanin-1 constructs were used as a negative control. The left panel show western blotting of the input cell extracts.

To map the C15ORF41-interacting domain on Codanin-1, U2OS cells were transiently transfected with bicistronic plasmids encoding GFP-C15ORF41 (full-length; aa1–281) and full-length RFP-Codanin-1, or RFP-Codanin-1 bearing segmental deletions (Figure 4A). Anti-GFP precipitates were subjected to western blotting with GFP or RFP antibodies; secondary antibodies conjugated to fluorophores were used so that GFP-tagged proteins on filters were red, and RFP-tagged proteins were green. As shown in Figure 4B (right panel), the smallest codainin1 fragment found to co-precipitate with C15ORF41 corresponded to the C-terminal 227 residues of Codanin-1 from glutamine residue at position 1000 to serine 1227. Fragments lacking this region of Codanin-1 were unable to interact with C51ORF41. Thus, the C-terminal 227 amino acids of Codanin-1 are necessary and sufficient for interaction with C15ORF41. We were unable to identify a subdomain of C15ORF41 responsible for interaction with Codanin-1 through deletion analysis as none of the fragments interacted with Codanin-1; as C15ORF41 is a small protein of 281 amino acids, truncations may simply destabilise the protein (data not shown).

We next tested if we could express the C15ORF41–Codanin-1 complex in bacteria. However, although different tagged forms of C15ORF41 could be expressed in bacteria in the soluble form at reasonable yields, we failed to express full-length Codanin-1 either alone or when co-expressed with C15ORF41 (data not shown). Consequently, we attempted to reconstitute C15ORF41 bound to a C-terminal fragment of Codanin-1. To this end, C15ORF41 with an N-terminal hexahistidine (His_6_) tag bearing the extra mutations V18M, C223S and W237R was expressed in bacteria, purified on Ni^2+^-NTA beads and eluted with imidazole (Supplementary Figure S2A). These extra mutations improved the solubility of C15ORF41 (J. Newman and O.Gileadi, personal communication). A nuclease-dead form of C15ORF41 (D^196^A) bearing the same solubilising mutations was also expressed. As shown in Supplementary Figure S1A, His_6_-C15ORF41 was expressed at reasonable yield, but preparations were consistently found to be contaminated with the bacterial metabolic enzyme ArnA. Next, a fragment of Codanin-1 (designated Codanin-1-C) corresponding to amino acids P800-S1227 was expressed in bacteria with an N-terminal hexahistidine tag, purified on Ni^2+^-NTA resin, eluted with imidazole and then the hexahistidine tag was cleaved off with TEV protease (Supplementary Figure S2B). To test the interaction of His_6_-C15ORF41 and Codanin-1-C, His_6_-C15ORF41 was re-bound to Ni-NTA beads, Codanin-1-C was passed over the beads, the proteins bound to these beads were eluted with imidazole, and the eluate was subjected to Superdex 200 size exclusion chromatography. A complex comprising C15ORF41 and Codanin-1-C could be seen clearly eluting from the Superdex column (Supplementary Figure S2C), with bacterial ArnA present as a contaminant. These data confirm that C15ORF41 interacts with the C-terminal region of Codanin-1.

### Impact of Codanin-1 on the cellular localisation of C15ORF41

**Figure 5.**
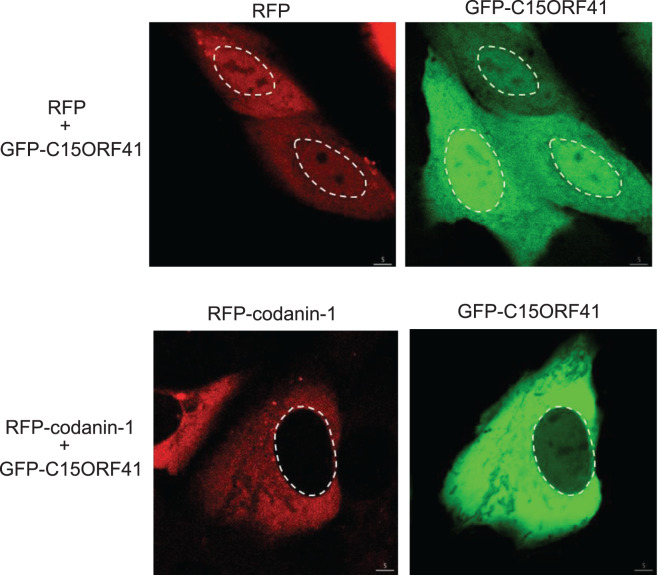
Impact of Codanin-1 on the cellular localisation of C15ORF41. U2OS cells stably expressing GFP-C15ORF41 were transfected with a plasmid expressing either RFP only or RFP-Codanin-1. Proteins were visualised through fluorescent imaging using a Zeiss 710 confocal microscope. The white dashed lines indicate the outline of the nucleus.

We investigated the cellular localisation of fluorescently tagged C15ORF41 and Codanin-1 when overexpressed in U2OS cells, as the antibodies currently available for these proteins do not appear to work in immunofluorescence experiments (data not shown). GFP-tagged C15ORF41 co-expressed with RFP alone was found in the cytoplasm as well as the nucleus. However, co-expression of GFP-tagged C15ORF41 with RFP-tagged Codanin-1 showed a different pattern of localisation, with C15ORF41 now found predominantly in the cytoplasm (Figure 5). Thus, the interaction with Codanin-1 may restrict C15ORF41 to the cytoplasm.

## Discussion

In this report, we demonstrated that C15ORF41 interacts with Codanin-1 when overexpressed, and also at the endogenous level; in this light, C15ORF41 has been designated the symbol CDIN1 for CDAN1 interacting nuclease 1 by the HUGO Gene Nomenclature Committee (HNGC). The interaction of the two proteins appears to be resistant to detergents and salt concentrations up to 1 M, indicating a robust interaction. The C-terminal 227 amino acids of Codanin-1 are necessary and sufficient for the interaction of the two proteins in cells, and recombinant, bacterially expressed C15ORF41 interacts with a C-terminal fragment of Codanin-1. Taken together these data show that the products of the only two known CDA-I genes (C15ORF41 and Codanin-1) interact physically in cells. The stoichiometry of interaction is not yet known, but the intensity of the endogenous Codanin-1 band seen in anti-GFP precipitates from cells overexpressing GFP-C15ORF41 suggest the interaction might be stoichiometric, or near-stoichiometric. From this point of view, C15ORF41–Codanin-1 might be considered a heterodimer. The mutations in C15ORF41 and Codanin-1 associated with CDA-I which we tested in this study have no apparent effect on the interaction with the partner subunit, suggesting that the mutations do not cause disease by disrupting the complex, although it could be argued that these mutations might affect nuclease activity instead. It will be interesting to profile other CDA-I mutations to check for disruption of the C15ORF41–Codanin-1 interaction.

Intriguingly, whereas C15ORF41 localises to the cytoplasm and nucleus, co-expression with Codanin-1 restricts C15ORF41 to the cytoplasm. This is reminiscent of the observation that Codanin-1 restricts histone supply in the nucleus by sequestering the histone chaperone ASF1. It will be interesting to test if the C15ORF41–Codanin-1 interaction is regulated in response to internal or external cues, as disrupting the complex would lead to the accumulation of C15ORF41 in the nucleus. This is particularly relevant given that C15ORF41 is predicted to be active as a nuclease, which might suggest a role in the nuclear compartment. Several PD-D/E-XK family nucleases are heterodimeric in nature, with both partners required for catalytic activities [20]. In this light, previous reports failed to detect nuclease activity associated with recombinant C15ORF41 alone [13], but we reasoned a partner protein — such as Codanin-1 — might be required for activity. We have carried out extensive testing for nuclease activity associated with recombinant forms of C15ORF41–Codanin-1, but failed to detect activity. Although we have been unable to express and purify C15ORF41 bound to full-length Codanin-1, instead we expressed C15ORF41 bound to a C-terminal fragment of Codanin-1 in bacteria (Supplementary Figure S1C). However, we failed to detect activity associated with this recombinant form of the complex towards a wide range of linear or branched radiolabelled DNA or RNA substrates, under a wide range of conditions with a range of different divalent cations (MS, JR, Anne-Cecile Declais and David Lilley, unpublished data). The substrates tested include circular DNA plasmids, linear single- and double-stranded DNA species made from synthetic oligonucleotides, and branched DNA structures including splayed duplexes, Y-forks, 5′ flaps, 3′ flaps — and Holliday junctions. We also tested activity towards linear RNA species and different RNA stem-loop structures (data not shown). Furthermore, we tested the GFP-C15ORF41–Codanin-1 complex affinity-purified from human cells, but we failed to obtain any evidence for nuclease activity. There are several potential explanations for failure to detect nuclease activity. First, the C-terminal fragment of Codanin-1 might not be sufficient to confer nuclease activity on the complex; full-length Codanin-1 may be required for nuclease activity. In this light, it would be interesting to test a range of higher eukaryotic Codanin-1 orthologues for full-length expression in bacteria and insect cells, since attempts to express and purify full-length Codanin-1 in this study failed (data not shown). Second, C15ORF41–Codanin-1 may have a very narrow substrate specificity, and the physiologically relevant substrate may not have been one of those tested in our study. Third, C15ORF41–Codanin-1 may require an activating post-translational modification missing from our bacterial preps. Fourth, the complex expressed in bacteria may not be folded properly because other subunits are missing. More work is needed to address these possibilities. One final possibility is that C15ORF41 is simply not a nuclease, despite predictions based on sequence, although this seems unlikely. More work will be required to test these possibilities. Structural analysis of the complex C15ORF41–Codanin-1 might also shed light on this mysterious complex.

## Data Availability

The original gel images can be found in the Supplementary Material.

## Footnotes

1 The construct for expressing recombinant C15ORF41^V18M C223S W237R^ was a kind gift from Joseph Newman, SGC, University of Oxford.

## Abbreviations

CDA: congenital dyserythropoietic anaemia
CDIN1: CDAN1 interacting protein 1
COPII: coat protein complex II
HBS: HEPES buffered saline
HtH: helix-turn-helix
RFP: red fluorescent protein
