# Supplementary material for: A complex comprising C15ORF41 and Codanin-1: the products of two genes mutated in congenital dyserythropoietic anaemia type I (CDA-I)
Source: Biochem J. 2020 May 28;477(10):1893–905. doi: 10.1042/BCJ20190944 (PMC7261414; doi:10.1042/BCJ20190944)

Figure 2A

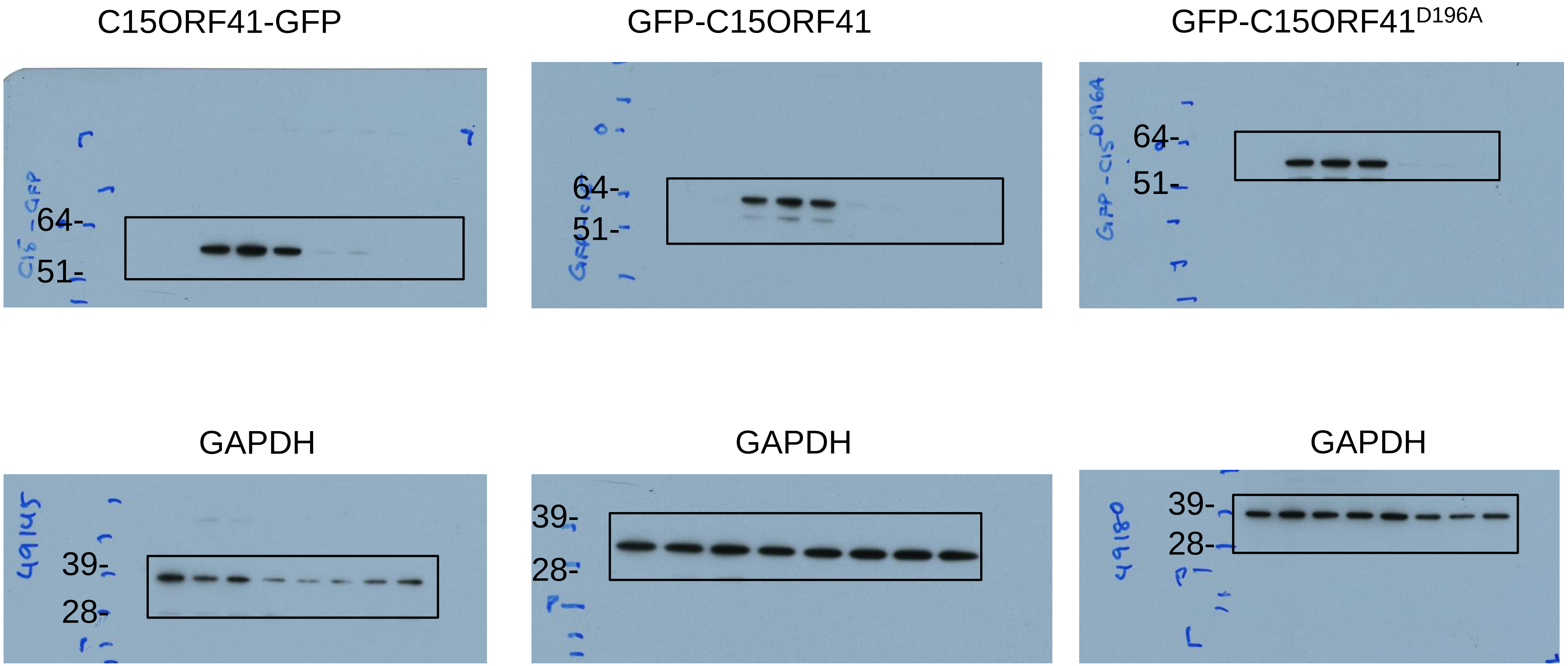

Developed using chemiluminescence and standard film

Figure 2B

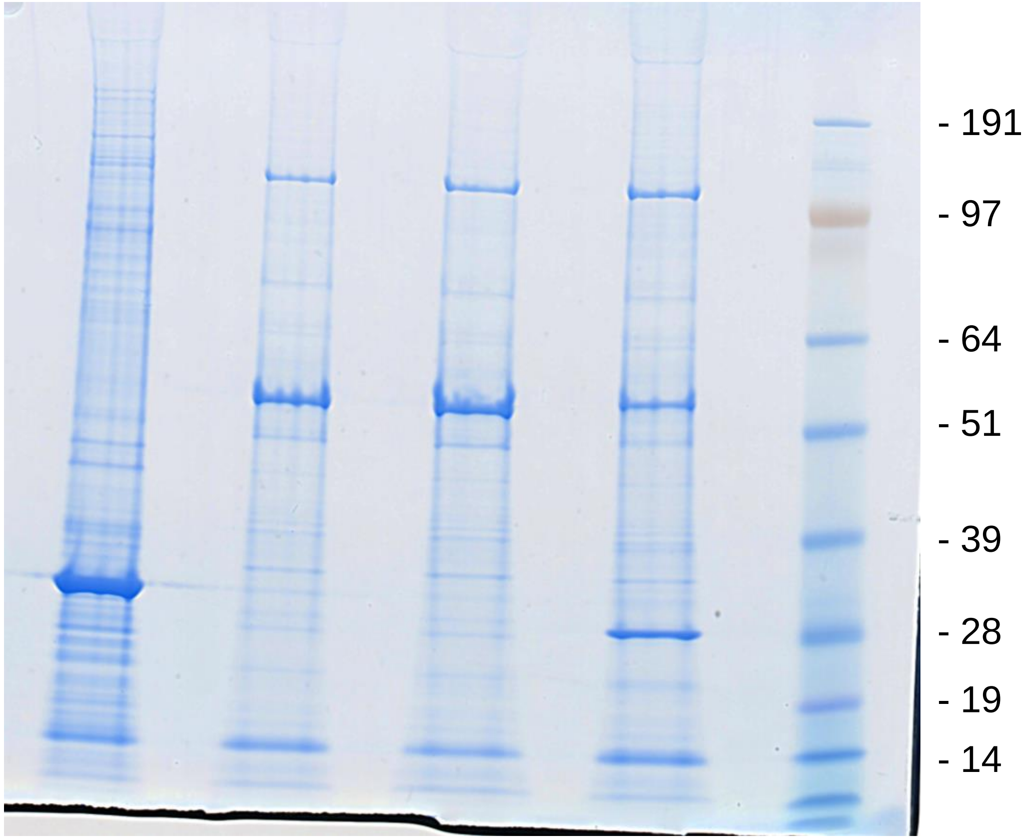

Figure 3A

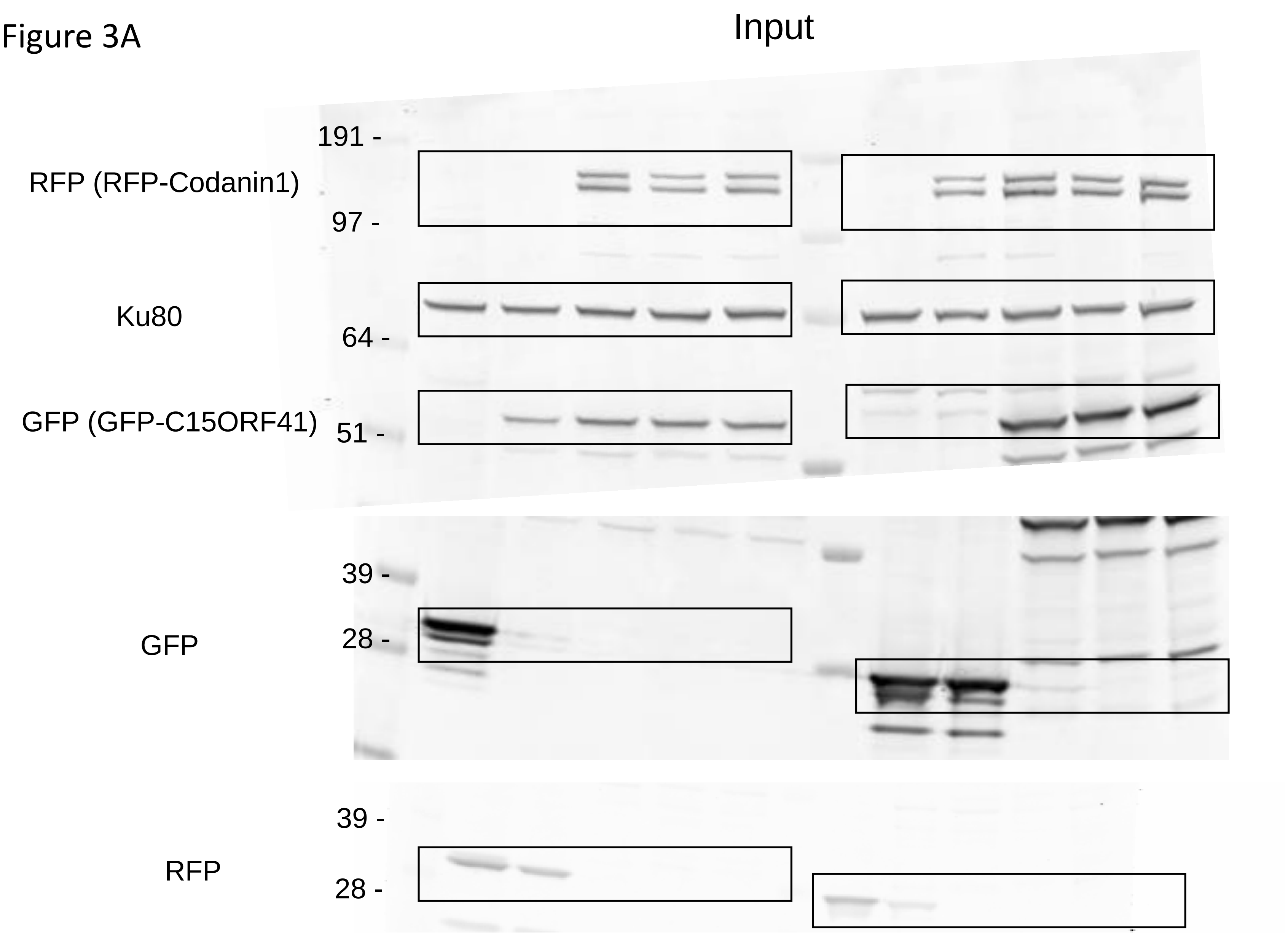

Developed using LiCOR to detect fluorescence

Figure 3A

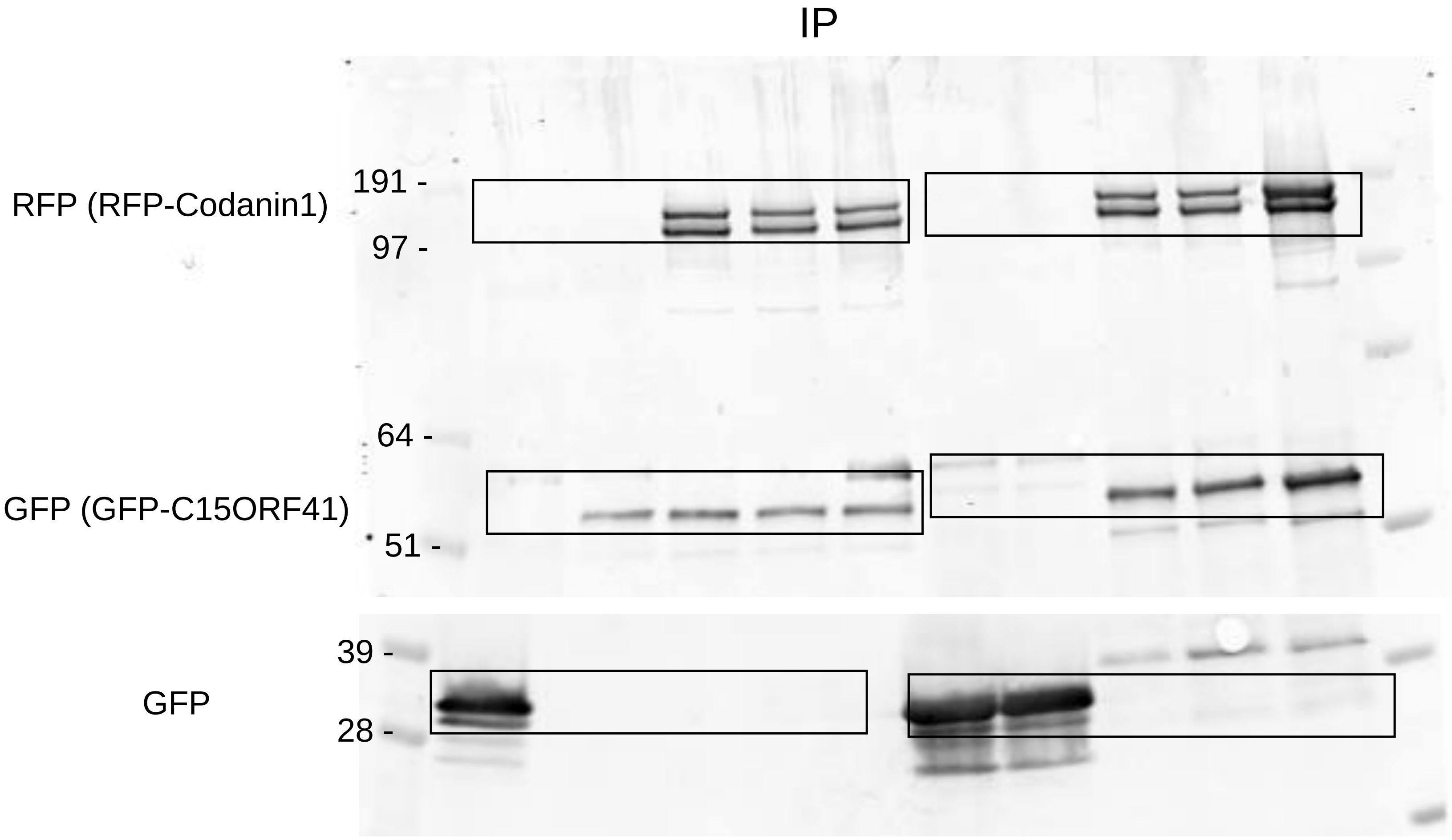

Developed using LiCOR to detect fluorescence

Figure 3B

C15ORF41

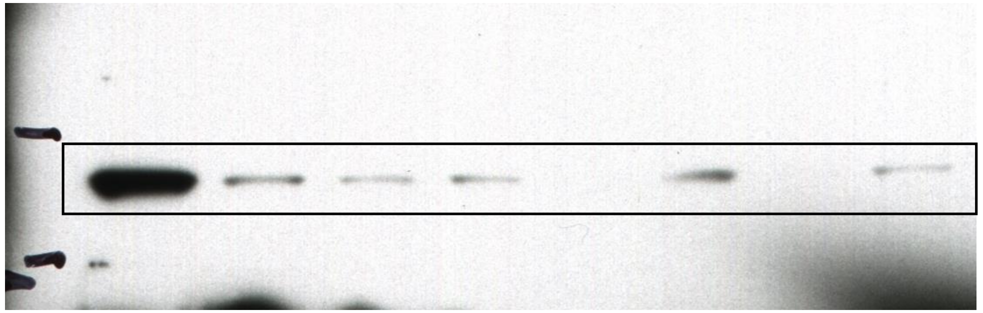

- 39

- 28

Developed using chemiluminescence and sensitive film

H3

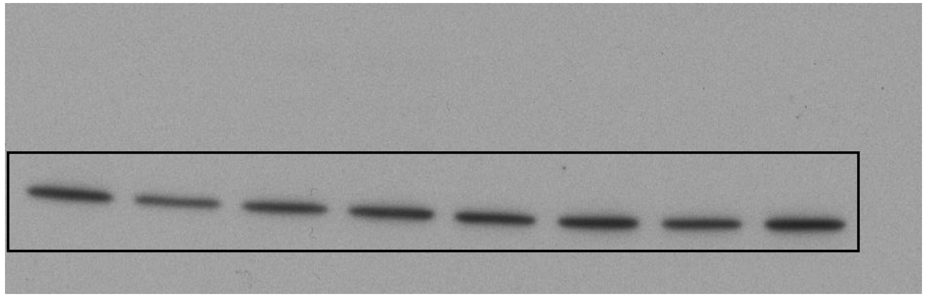

- 19

- 14

Developed using chemiluminescence and standard film

Figure 3C

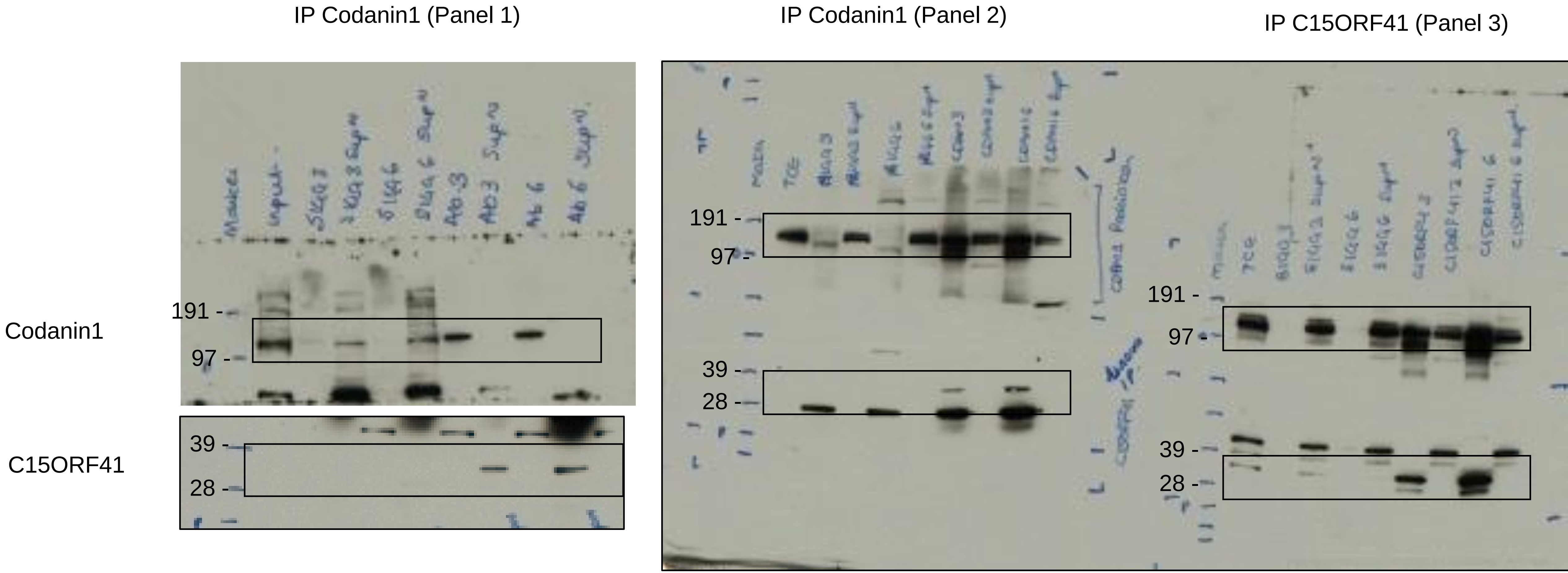

Developed using chemiluminescence and standard film

Figure 3D

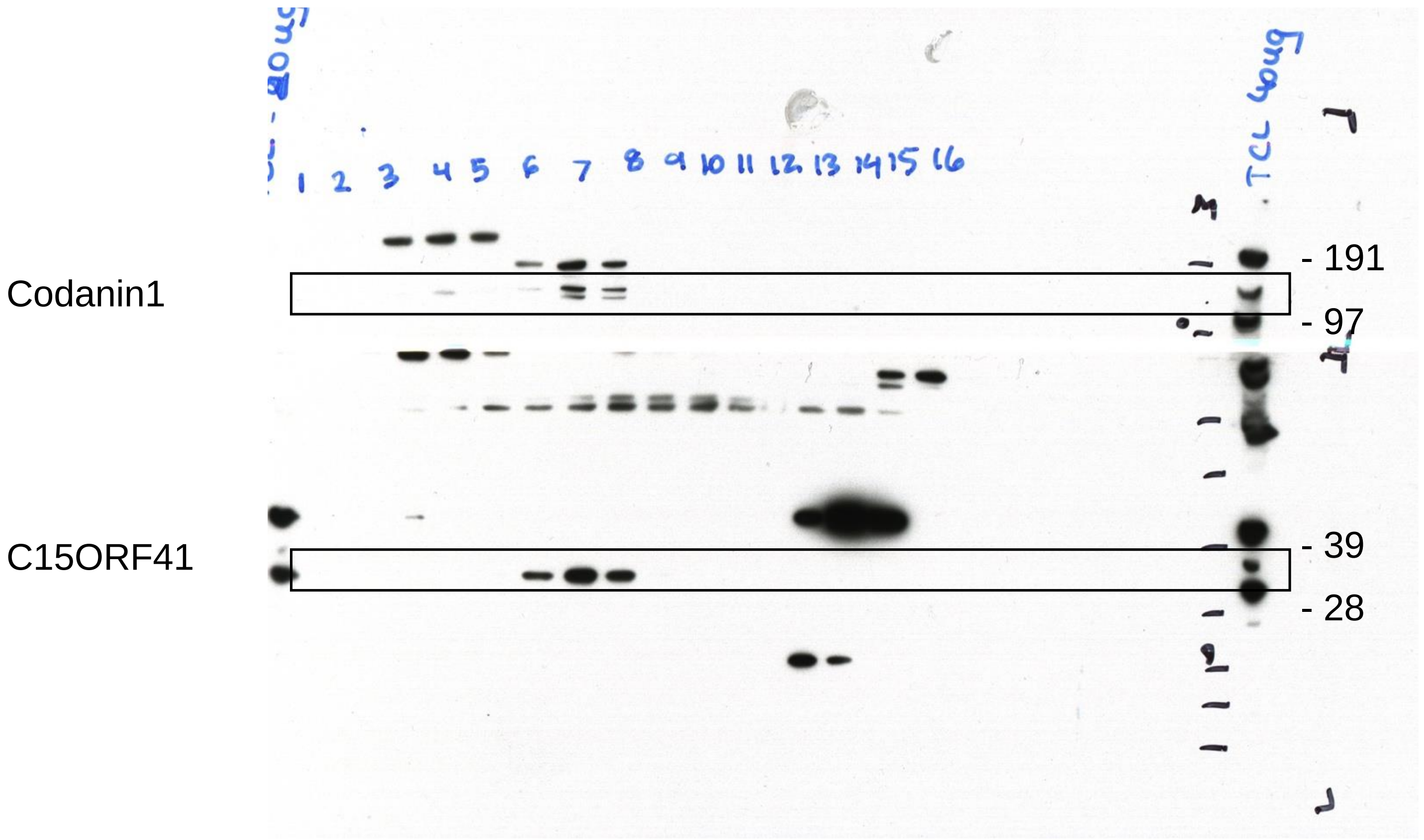

Developed using chemiluminescence and sensitive film

Figure 4B

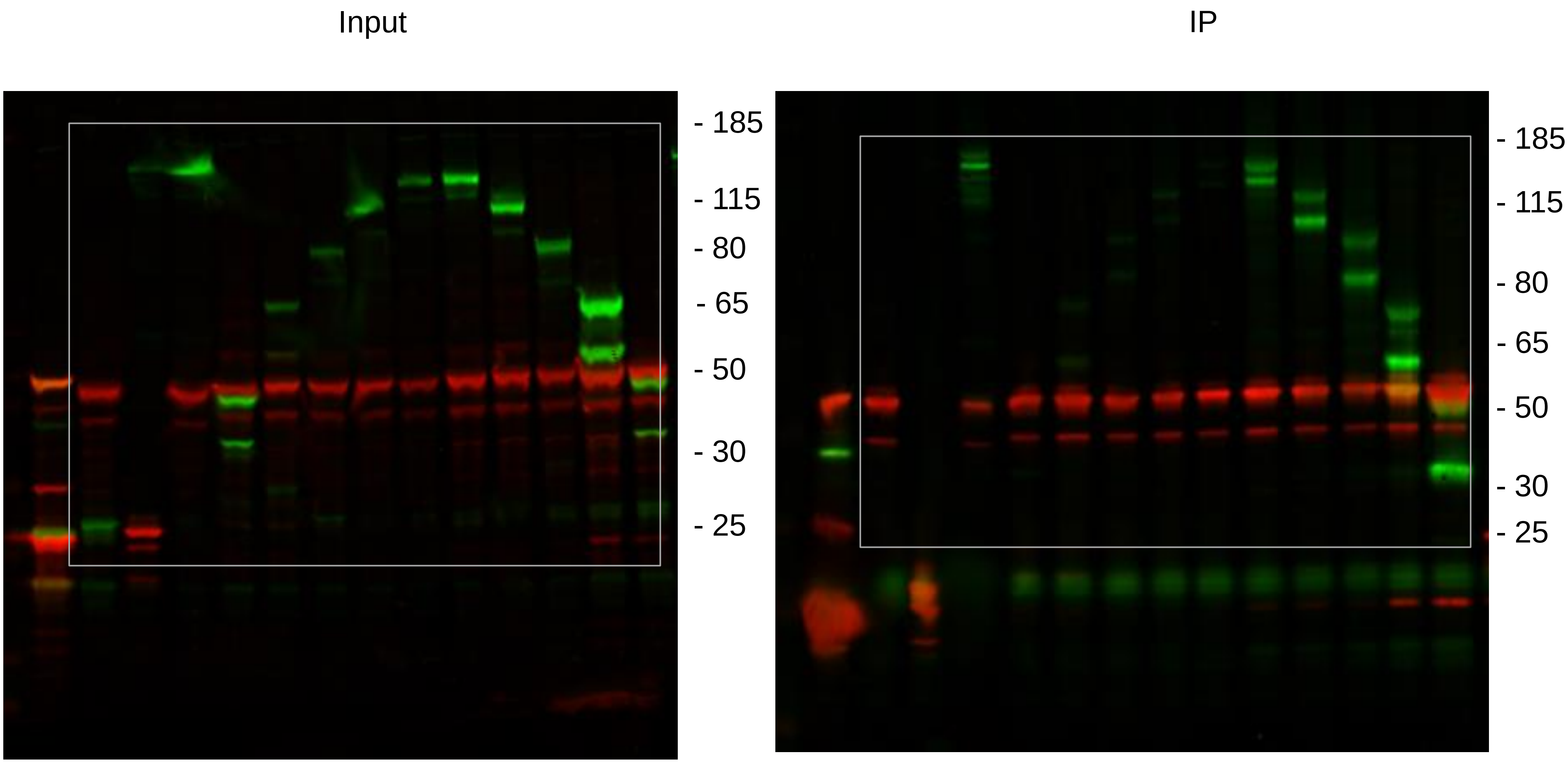

Developed using LiCOR to detect fluorescence

Figure S1A

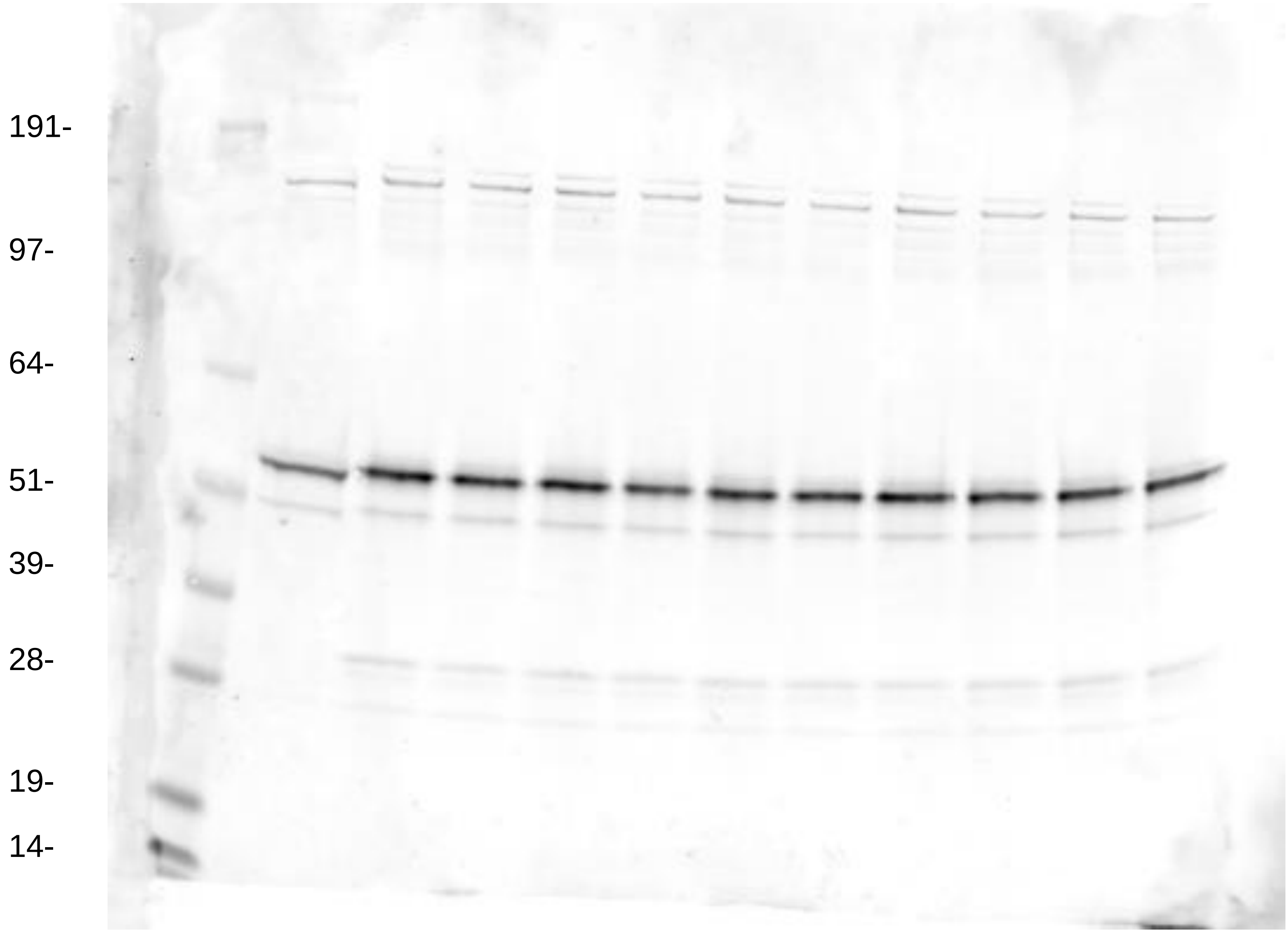

Developed using LiCOR to detect fluorescence

Figure S1B

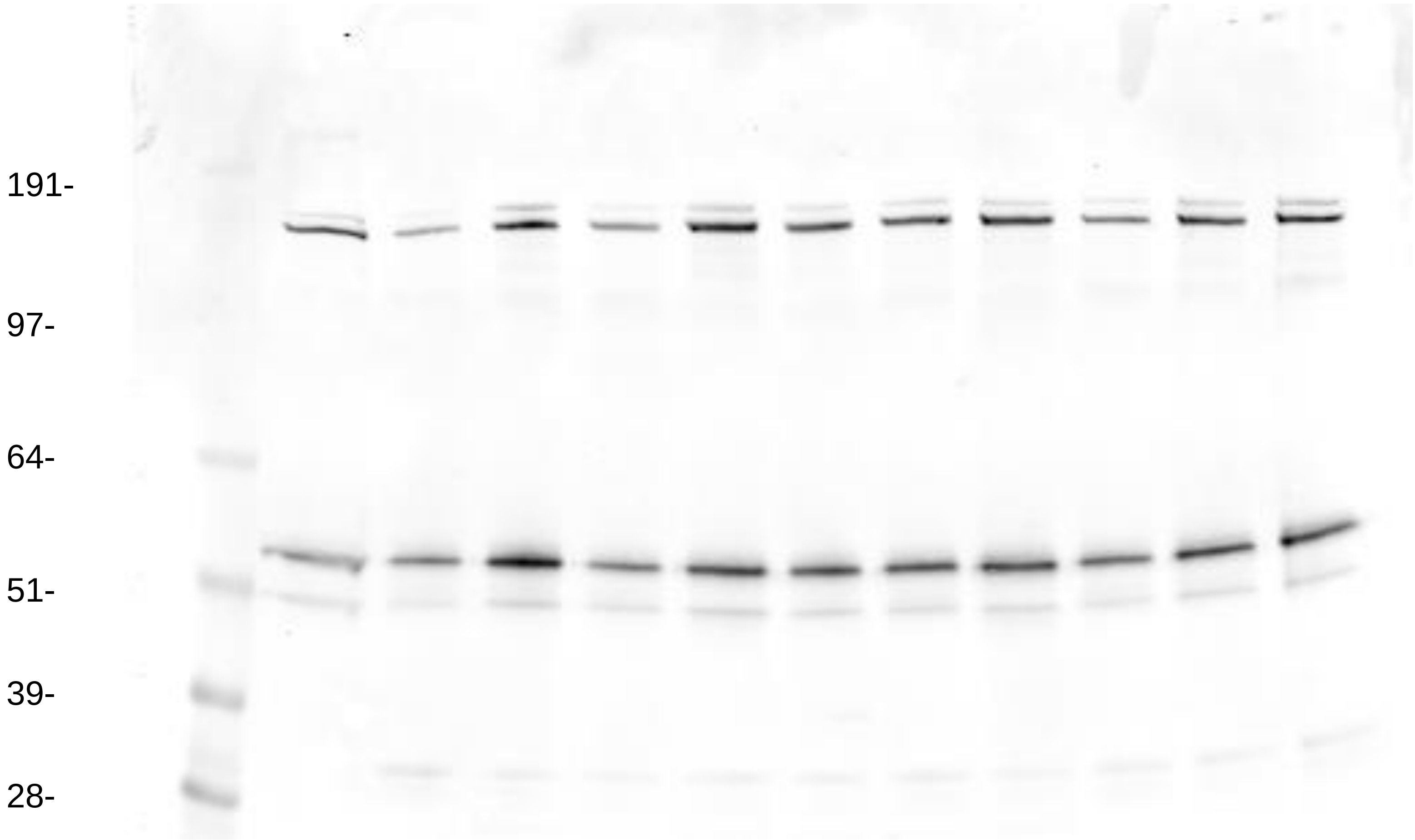

Developed using LiCOR to detect fluorescence

Figure S2A

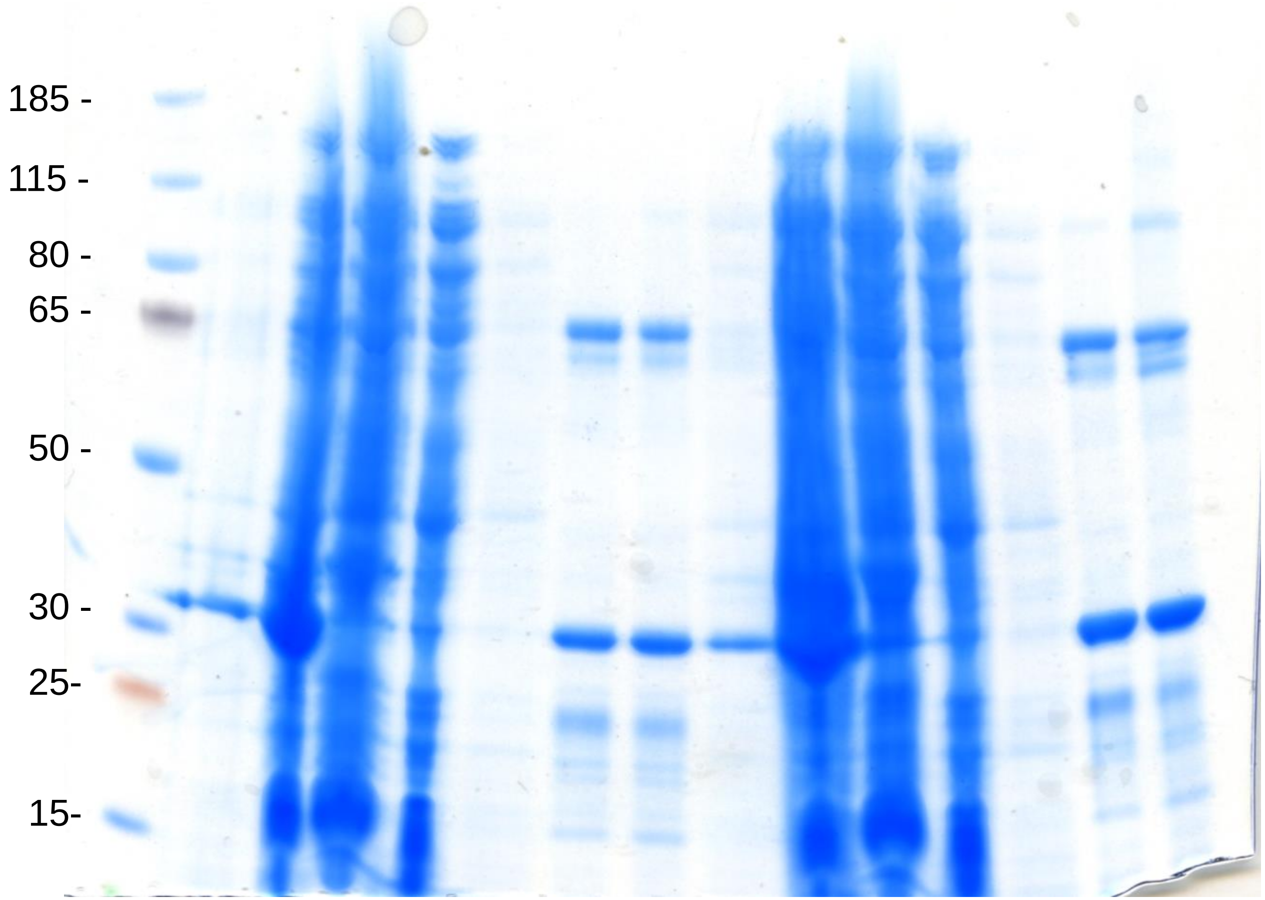

Figure S2B

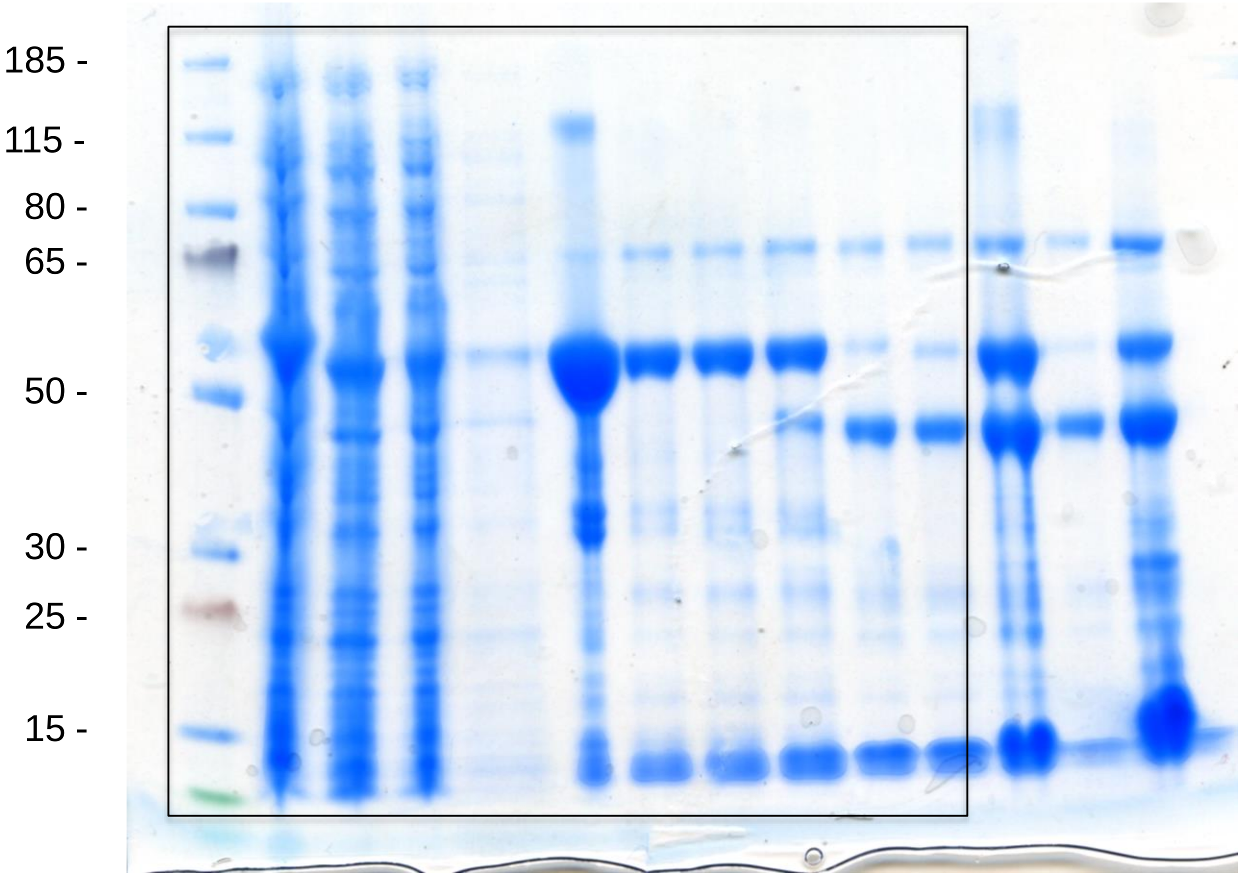

Figure S2C

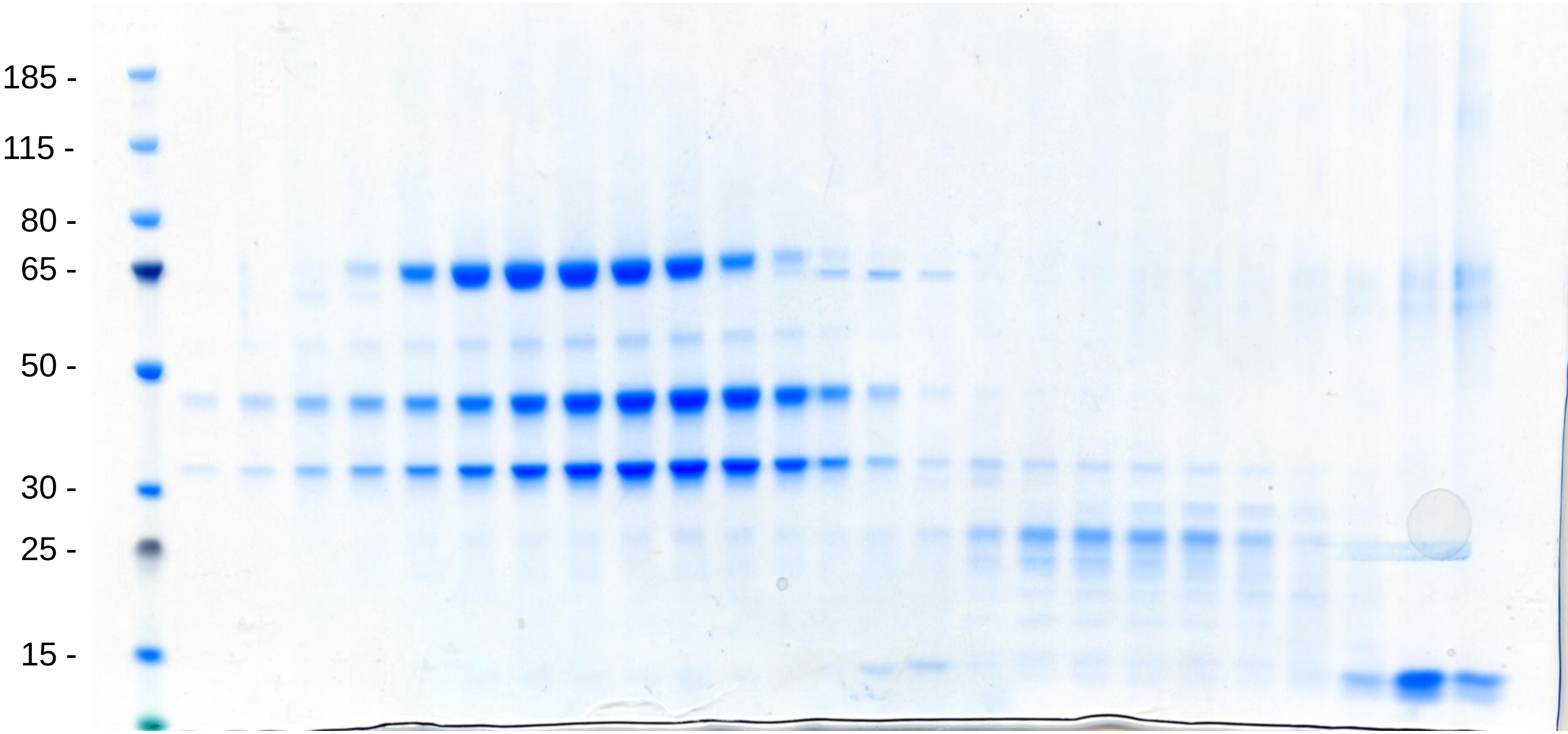

Supplement: Supplementary Figures S1-S4 [file BCJ-477-1893-s1.pdf]
